# Comparison of Carbon-Nanoparticle-Filled Poly(Butylene Succinate-co-Adipate) Nanocomposites for Electromagnetic Applications

**DOI:** 10.3390/nano12203671

**Published:** 2022-10-19

**Authors:** Miks Bleija, Oskars Platnieks, Jan Macutkevič, Olesja Starkova, Sergejs Gaidukovs

**Affiliations:** 1 Institute of Polymer Materials, Faculty of Materials Science and Applied Chemistry, Riga Technical University, P. Valdena 3/7, LV-1048 Riga, Latvia; 2 Faculty of Physics, Vilnius University, Sauletekio 9, LT-10222 Vilnius, Lithuania; 3 Institute for Mechanics of Materials, University of Latvia, Jelgavas 3, LV-1004 Riga, Latvia

**Keywords:** graphene nanoplatelets, multi-walled carbon nanotubes, nanostructured carbon black, amorphous carbon black, electrostatic dissipative, electromagnetic interference shielding, anti-static, electrical conductivity, thermal conductivity, mechanical properties

## Abstract

Electrostatic dissipative (ESD), anti-static (AS), and electromagnetic interference (EMI) shielding materials are commonly based on commodity fossil-fuel-based plastics. This, in turn, contributes to ever-growing non-biodegradable plastic pollution. Graphene nanoplatelets (GN), multi-walled carbon nanotubes (MWCNT), nanostructured carbon black (NCB), and amorphous carbon black (CB) were utilized as nanofillers to prepare bio-based and biodegradable poly(butylene succinate-co-adipate) (PBSA) nanocomposites. Solvent-cast composites were prepared with 1.1 to 30.0 vol.% nanoparticle loading. The literature mainly focuses on relatively low loadings; therefore, for this research, filler loadings were increased up to 30 vol.% but the maximum loading for NCB and CB loadings only reached 17.4 vol.% due to a lack of dimensional stability at higher loadings. The composites were characterized using tensile testing, volumetric and surface conductivity measurements, thermal conductivity measurements, dielectric spectroscopy in the microwave region, and transmittance in the terahertz range. Tensile tests showed excellent carbon filler compatibility and enhanced tensile strength for loadings up to 5 vol.% (up to 20 vol.% for MWCNT). The highest thermal conductivity values were reached for the MWCNT filler, with the 30.0 vol.% filled composite reaching 0.756 W/mK (262% increase over PBSA). All fillers were able to produce composites that yielded volume conductivities above 10^−10^ S/m. Composites with MWCNT, GN, and NCB inclusions above the percolation threshold are suitable for EMI applications in the microwave and THz frequency range.

## 1. Introduction

With the rising prevalence of electronics and technology in modern life and society, waste generation during the manufacturing, exploitation, and disposal of devices and materials is emerging as a serious challenge. [1,2,3,4]. Plastics are widely used as packaging materials due to their versatility, low cost, low density, and adaptability. However, they constitute the majority of non-biodegradable consumer waste [5,6]. Electronic devices and components require electrostatic dissipative (ESD) and anti-static (AS) materials both in transport and storage and in use. The manufacturing of electronic components grows year after year [7]. It is one of the potential industries where biodegradable and bio-based polymers may play a significant role in reducing environmental impact. Carbon nanoparticles are perspective fillers to achieve ESD and AS properties, as they enable the creation of electrically and thermally conductive polymer composites due to their unique properties and the formation of percolated networks at relatively low concentrations [8,9]. Research is required before the industry can adapt to biodegradable replacements.

Depending on the application of the ESD material, different types of plastic can be used. For packaging individual components, polymers such as low-density polyethylene (LDPE) and polyethylene terephthalate (PET) are frequently used as blown films and bags. For rigid bulk packaging, thermoformed or blow-molded polystyrene (PS), PET, and poly(ethylene terephthalate-glycol) are often used. Rigid onboard components or parts in devices can be injection molded from polyamides (PA), acrylonitrile-butadiene-styrene (ABS), and other performance polymers. Biodegradable polymers as matrices for ESD materials are relatively new, and most research is still done on synthetic polymers [10,11,12,13]. From biodegradable polymers, polylactic acid (PLA) has drawn the most attention both in research and commercialization. However, PLA is quite rigid, and therefore it is limited to extrusion, fused filament fabrication, and injection molding. Poly(butylene succinate-co-adipate) (PBSA) is biodegradable, partially bio-based, and, according to the manufacturer’s specification, depending on the grade, it can be blown as films or injection-molded as rigid parts [14]. PBSA has exceptional potential as an alternative to LDPE in the aspect of mechanical properties [15] as well as its relatively higher electrical conductivity [16].

The addition of carbon nanoparticles stands out as a simple and compatible solution for modifying polymer properties, while maintaining desirable properties, such as mechanical strength, chemical resistance, and low density. The properties of these composites widely depend on the nanoparticles used, filler concentration, and processing methods [17]. Carbon black (CB) is relatively cheap and is widely used for UV protection on an industrial scale. CB can also enhance electrical conductivity; however, quite a high filler loading level is required to achieve sufficient improvement. A study by da Silva et al. attained electrically conductive PLA/CB composites at filler loadings above 10 wt.% [18]. Nanostructured carbon black (NCB) offers enhanced electrical and thermal conductivity in addition to mechanical enhancement at lower filler concentrations. NCB has been applied to elastomeric matrices to attain enhanced strain-dependent conductivity at concentrations of 5 to 10 wt.% [19,20,21,22]. Yuan et al. achieved a percolation threshold of 1.5 vol.% for NCB in polypropylene [23]. The most promising and widely researched nanofillers for the enhancement of electrical properties are multi-walled carbon nanotubes (MWCNT) and graphene nanoplatelets (GN). CNTs can easily form percolated networks due to their extremely high aspect ratios, which yield high electrical and thermal conductivity [24,25]. Socher et al. achieved a percolation threshold at 0.7 wt.% for MWCNT in polyamide-12 [26]. GN has very high in-plane electrical and thermal conductivity (~5000 W/mK) [27,28]. Shtein et al. reported an epoxy-GN composite that reached a thermal conductivity of 12 W/mK at 25 vol.% due to the uniform orientation of nanoparticles and a defect-free structure, as well as an electrical percolation threshold at 5 vol.% [29]. In addition, GN offers improved rheological and barrier properties due to platelet morphology, which allows slippage and high packing factors [30].

Research into PBSA-based ESD and AS materials in the literature is very scarce, but there have been some reports about poly(butylene succinate) (PBS), which shows somewhat similar properties to PBSA. Shih et al. reported the preparation of biodegradable PBS/MWCNT nanocomposites, achieving a nine order-of-magnitude increase in electrical conductivity and a 120% increase in storage modulus at 3 wt.% filler loading, thus enabling the usage in ESD applications [31]. Lin et al. demonstrated an eleven order-of-magnitude increase in electrical conductivity and a 113% increase in storage modulus at 3 wt.% filler loading in a PBS using surface-modified MWCNT, and also proposed this material for ESD and AS applications [32]. Other studies have investigated PBS and carbon nanoparticle composites and their electrical properties, while not venturing in depth into the ESD performance. Shi et al. achieved improved electromagnetic interference (EMI) shielding properties for PBS, polyurethane, and 2.5 wt.% MWCNT segregated composite [33]. A study by Wang et al. observed a five order-of-magnitude increase in electrical conductivity for a PBS nanocomposite loaded with 2 wt.% GN [34]. In our previous study, a five order-of-magnitude increase in electrical conductivity was achieved for a PBS loaded with 6 wt.% GN, while lower concentrations did not show electrical percolation [30].

In this paper, we compare 18 solvent-cast PBSA composites filled with four different carbon-based nanofillers with loading from 1.1 to 30.0 vol.%. The composites were characterized using tensile testing, volumetric and surface conductivity measurements, thermal conductivity measurements, dielectric spectroscopy in the microwave region, and transmittance in the terahertz range. PBSA was selected for this study as a promising bio-based, biodegradable (industrially compostable), and environmentally sustainable polymer matrix for use as an alternative to commodity plastics in ESD and AS packaging applications.

## 2. Materials and Methods

### 2.1. Materials

All the materials used in this work are available commercially. Poly(butylene succinate-co-adipate) (PBSA) FD92PM pellets were purchased from PTT MCC Biochem Co., Ltd., (Bangkok, Thailand). PBSA is a semi-crystalline thermoplastic polyester with a density of 1.24 g/cm^3^ and MFI of 4 g/10 min (190 °C, 2.16 kg). It is completely biodegradable and partially bio-based (20–50%, DIN certification 8C083). Multi-walled carbon nanotubes (MWCNT) NC7000 (density 1.85 g/cm^3^, aspect ratio (l/d) of 158) were purchased from Nanocyl SA, (Sambreville, Belgium). Exfoliated graphene nanoplatelets (GN) xGnP-C-750 (density 2.13 g/cm^3^, l/d 2000) were purchased from XG Sciences, Inc. (Lansing, MI, USA). Nanostructured carbon black (NCB) Printex XE-2B (density 1.8 g/cm^3^, average particle size 30 nm) was purchased from Orion Engineered Carbons GmbH (Cologne, Germany). Amorphous carbon black (CB) (density 1.8 g/cm^3^, average particle size 150 nm) was purchased from US Research Nanomaterials, Inc., (Houston, TX, USA). Chloroform was purchased from Merck KGaA (Darmstadt, Germany). PBSA pellets were dried under vacuum in a vacuum drying oven (J.P. Selecta, Barcelona, Spain) before use, according to the manufacturer’s recommendation (70 °C, 24 h). Carbon fillers were used as received without any further purification and stored in sealed packaging.

### 2.2. Sample Preparation

The nanocomposites were prepared via the solvent-casting process. The carbon fillers were dispersed in chloroform using an ultrasonic sonotrode Hielscher UIS250V (Hielscher Ultrasonics GmbH, Teltow, Germany) for 15 min. Afterward, PBSA pellets were dissolved in chloroform and combined with the nanoparticle dispersion, then homogenized using the ultrasonic sonotrode for 15 min, followed by further homogenization with a high-shear mixer Silverson L5M-A (Silverson Machines Ltd., Chesham, UK) at 10,000 RPM for 30 min. The resulting solution was cast in a Petri dish and left in a fume hood overnight for chloroform to evaporate. Any leftover solvent was removed by vacuum drying in a vacuum drying oven for 24 h at 70 °C. The filler weight (wt.%) and volume (vol.%) fractions of the produced systems can be seen in Table 1. The maximum loading of GN and MWCNT was 30.0 vol.%, while in the case of NCB and CB it was only 17.4 vol.% due to filler agglomeration, which resulted in brittle composites. This also limited the maximum loading for the tensile testing to 8.3 vol.% for NCB and 7.2 vol.% for CB.

Solvent-cast samples were cut into pellets. To obtain precise sample dimensions, compression molding (135 °C, 5 min) followed by rapid cooling between steel plates to room temperature was used. Composite films (with a thickness of 100 μm, 500 μm, and 700 μm) were prepared for testing.

### 2.3. Characterization

Interventional Uniaxial tensile tests were performed on a 2.5 kN universal testing machine (ZwickRoell GmbH and Co., KG, Ulm, Germany) at a constant crosshead speed of 2 mm/min according to ASTM D882 with a 0.2 N pre-load. Tabs made from a paper tape were applied to samples to ensure smooth loading and prevent sample slippage during the test. Tests were performed for five replicate samples for each composition. The secant elastic modulus was determined in the strain range of 0.2–0.5%.

Volume conductivities of the PBSA composites were obtained using a broadband dielectric spectrometer (DS) Novocontrol BDS 50 (Novocontrol Technologies GmbH and Co., KG, Montabaur, Germany). Disc-shaped samples with a thickness of 0.5 mm and a diameter of 30 mm were placed between plate electrodes and measured at room temperature with the electrical field frequency set to 1 Hz.

The sheet resistance of the PBSA composites was determined using a four-point Signatone S-302-4 probe (Signatone Corporation, Gilroy, CA, USA) and electrical source meter unit Keithley 2450 (Keithley Instruments, LLC, Cleveland, OH, USA). The square-shaped films with a thickness of 100 μm and an edge length of 60 mm were tested in a current range of 10 nA to 10 mA with a maximum voltage of 21 V until the measured resistance value stabilized. Measurements were repeated five times for each sample.

The sheet resistance was calculated according to a manual by Signatone and is as follows:

(1) $$R_{s}=4.5324\frac{U}{I}f_{1}f_{2}$$

where $R_{s}$ is the electrical sheet resistance (Ω/sq), 4.5324 is the correction factor determined by the model of the four-point probe according to the technical data by Signatone, U is the measured voltage (V), I is the applied current (A), $f_{1}$ is the correction factor for the sample thickness, and $f_{2}$ is the correction factor for the sample size. ($f_{2}$ = 1 as the probe spacing is magnitudes of order smaller than the sample size).

The correction factor for sample thickness is calculated as follows:

(2) $$f_{1}=\frac{\ln\left(2\right)}{\ln\left[\frac{\sinh\left(\frac{t}{s}\right)}{\sinh\left(\frac{t}{2s}\right)}\right]}$$

where t is the thickness of the sample (100 μm) and s is the probe spacing (1000 μm).

Thermal diffusivity measurements were obtained using a light-flash apparatus LFA 447 NanoFlash (NETZSCH-Gerätebau GmbH, Selb, Germany) according to EN ISO 22007-4. The square shaped samples with an edge length of 12.7 mm and a thickness of 0.7 mm were spray coated with a graphite-based coating Graphit 33 (Kontakt Chemie, Zele, Belgium) to ensure the equal opacity and absorbance of the samples. Heat capacities of the samples were obtained by comparison to a Pyrex sample with a known heat capacity. The samples were subjected to five repeated thermal diffusivity measurements at three temperatures 25, 35 and 45 °C.

The densities of the PBSA composites were determined using the hydrostatic displacement method by measuring sample weight in air and in ethanol on analytical scales Sartorius KBBA 100 (Sartorius AG, Göttingen, Germany) equipped with a Sartorius YDK 01 hydrostatic density measurement kit. Density (d (g/cm^3^)) was calculated according to the following equation:

(3) $$d=\frac{m_{a}\left(d_{\mathrm{EtOH}}-0.0012\right)}{0.99983\left(m_{a}-m_{s}\right)}+0.0012$$

where $m_{a}$ (g) is the sample’s mass in air, $m_{s}$ is the sample’s apparent mass measured submerged in ethanol (g), and $d_{\mathrm{EtOH}}$ is the density of ethanol (0.805 g/cm^3^), determined with a hydrometer.

Thermal conductivity (λ (W/mK)) was calculated according to the following equation:

(4) λ(T)=a(T)d(T)Cp(T)

where a is thermal diffusivity (mm^2^/s), d is the sample density (g/cm^3^), Cp is the sample specific heat capacity (J/(gK)), and T is the absolute temperature (K).

The thermal conductivity activation energies ($E_{a}$ (eV)) of the samples were calculated according to the Arrhenius equation [35]:

(5) $$\lambda =\lambda_{0}e^{\left(-\frac{E_{a}}{kT}\right)}$$

where λ is the thermal conductivity (W/mK), $\lambda_{0}$ is the extrapolated inherent thermal conductivity at infinite temperature (W/(mK)), T is absolute temperature (K), and k is the Boltzmann constant (8.617 × 10^−5^ eV/K).

In the microwave frequency range from 24 to 40 GHz, a home-made thin rod waveguide spectrometer as described in a previous study [36] was used. Cylindrically shaped samples with a thickness of 0.5 mm were placed in waveguide holder. The measurements are determined to be accurate to within 10%.

In the terahertz frequency range from 0.01 to 3 THz, a terahertz time domain spectrometer (EKSPLA UAB, Vilnius, Lithuania) based on a femtosecond laser (1 µm wavelength, pulse duration under 150 fs) was used for the spectrum measurements. A photoconductive terahertz emitter-detector based on GaBiAs was used. The noise-to-signal ratio was the highest (60 dB) at 0.5 THz, where the accuracy was to within 1%. The thickness of all samples for THz investigations was 0.5 ± 0.1 mm.

## 3. Results and Discussion

### 3.1. Tensile Properties

**Figure 1:**
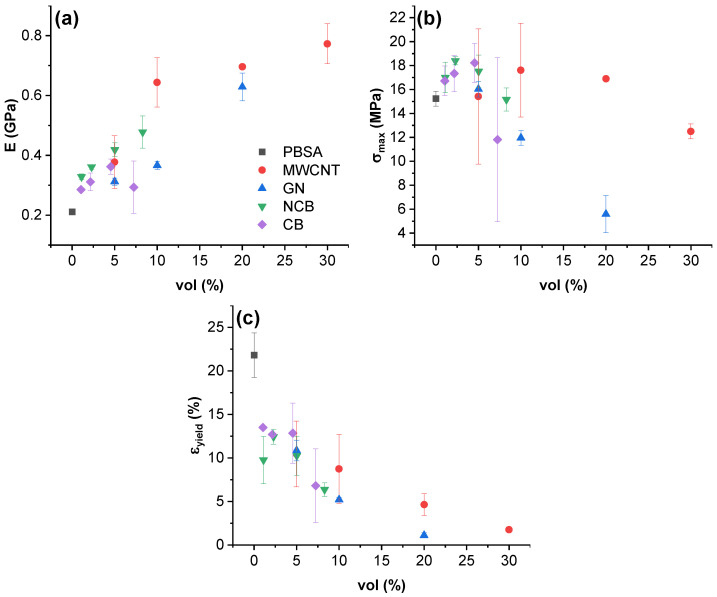
Tensile properties of PBSA/carbon nanoparticle composites: (**a**) elastic modulus, (**b**) tensile strength, and (**c**) yield elongation.

Figure 1 shows elastic modulus, tensile strength (yield stress), and elongation at yield for PBSA/carbon nanoparticle composites with different filler concentrations. Representative stress–strain curves are shown in Supplementary Materials (Figure S1). With the increase in concentration of carbon nanoparticle fillers, a transition from a ductile to a brittle mechanical response can be observed as an increased elastic modulus and decreased yield elongation values. Neat PBSA shows ductile deformation with extensive necking and failure in a plastic manner (Figure S1). The tested compositions with MWCNT, GN and 8.3 vol.% NCB showed a clear transition to a brittle failure mode. The 17.4 vol.% NCB, 17.4% CB, and 30.0 vol.% GN composites were too brittle for tensile tests. With exception of CB, the elastic modulus increased with the carbon nanoparticle loading. MWCNT-filled composites displayed the highest increases in elastic modulus (from 79% to 266%), followed by GN filled composites (from 48% to 198%), and NCB-filled composites (from 56% to 127%). Comparatively, NCB addition was most sensitive to increases in elastic modulus at low concentrations from 1.1 to 5.0 vol.%, while MWCNT contributed to significant increases at high loadings from 10.0 to 20.0 vol.%.

For MWCNT-filled composites, tensile strength showed improvements at 5.0 to 20.0 vol.% (up to 16% compared to neat PBSA) concentrations, while a slight drop was observed at 30.0 vol.% loading. GN, however, showed only a 5% improvement over neat PBSA at a 5.0 vol.% concentration, but higher concentrations of filler severely reduce the tensile strength. For NCB-filled composites, tensile strength increased by up to 21% compared to neat PBSA, which can be seen at 1.1–5.0 vol.% NCB loadings, but higher loadings displayed no improvement. A similar trend can be observed in CB-filled composites, with tensile strength increasing by up to 20%, reaching maximum at a concentration of 4.5 vol.%, while above this concentration a significant decrease and inconsistent values are observed, further indicated by the error bars. It has been reported that under a critical concentration, spherical fillers act as crack arresters [37], while above as stress concentrators that aid in crack initiation and propagation [38]. Likewise, filler geometry also has an impact on the stress-transfer properties, where planar fillers such as GN would likely induce increased matrix stresses near the thin edges of the platelets. In addition, the high specific surface of GN would significantly restrict polymer-chain movements, while MWCNT’s linear structure could better entangle with long polymer chains. 

The addition of every filler reduces yield elongation value by around 50% or more. This could be explained by the local shear instability of a crystal lattice caused by introduced material heterogeneity, thus plastic deformation, i.e., chain slippage, which is characteristic for neat PBSA, is disturbed [39]. The lowest yield elongation can be seen for the 20.0 vol.% GN-filled and 30.0 vol.% MWCNT-filled composites. However, MWCNT composites above 5.0 vol.% loading showed the highest elongation values compared to other fillers, and this could be attributed to the high aspect ratio of MWCNT allowing them to halt crack propagation by bridging them [40]. 

The tensile properties of carbon-nanoparticle-reinforced polymer composites have been widely investigated and discussed in scientific literature, with CNT and GN being the most prevalent fillers. However, literature regarding the usage of PBS or PBSA as a polymer matrix is somewhat scarce. Biodegradable polyesters such as PLLA [41,42,43,44] as well as regular synthetic polymers such as polyolefins [45,46,47], polyamides [48] and polyurethanes [49] are more widely researched as polymer matrices in carbon-filled composite applications. In a study by Alruwail et al., a PBS/PLA blend composite filled with MWCNT was developed for gas-separation-membrane applications [50]. For a 0.5 wt.% filled composite, a 30% increase in tensile strength and a 13% increase in elastic modulus were achieved. The effect was attributed to a strong interaction between the PBS/PLA matrix and the CNTs. Chopra et al., reported a 12% increase in tensile strength and a 21% increase in elastic modulus in a polyamide-6 with the addition of 0.5 wt.% MWCNT composite, achieving a good level of dispersion and distribution, thus promoting more efficient stress transfer to the filler and enhanced mechanical properties [48]. Our results match the literature, indicating the excellent mechanical reinforcing capabilities of CNTs. Snowdon et al. investigated melt-processed PBS and carbon-black-filled composites at loadings of 1, 3 and 5 wt.% and demonstrated a 12% increase in elastic modulus, 5% increase in tensile stress, and a 10% decrease for elongation at break. The interaction between the filler and the matrix was estimated by calculating the composite-matrix tensile yield strength ratio, which was constant at 1.05, and indicated good interfacial adhesion [51]. This matches well with our observations, indicating a good filler distribution and synergetic properties in composites with loadings of up to 4.5 vol.%. Extruded high-density polyethylene reinforced with 1, 2 and 3 wt.% graphene nanoplatelets showed no improvement in the tensile strength compared to a neat matrix, as well as a greatly reduced plastic plateau, which could not be seen in the 3 wt.% filled composite. Seretis et al. ascribe this effect to the formation of pores for increasing GN content that act as stress concentrations and embrittle the composite. GN does not contribute to the strengthening before the plastic region due to slippage between individual nanoplatelets [52]. Indeed, higher loadings for GN filler seem to significantly reduce mechanical properties, again matching well with our observations for PBSA/GN composites. Finally, nanostructured carbon black, also known as conductive carbon black, is mainly used in elastomer matrices such as polyisoprene [53], acrylonitrile-butadiene [54], and polysiloxane [55]. However, a paper by Liu et al. describes the incorporation of Printex XE-2 in a PLLA matrix via melt-processing and further characterization. The study found increased tensile strength for composites with filler loading up to 2 wt.% compared to a neat matrix. Composites with filler content up to 10 wt.% showed greatly enhanced elastic modulus while sustaining adequate tensile strength. Composites with high loadings above a 10.0 vol.% displayed diminishing returns for the enhancement of mechanical strength, which is speculated to be caused by the agglomeration of filler particles, thus causing stress concentrations and crack initiation [56]. The use of high loadings was aimed at electrical properties, which are discussed in the following sections. In our case, the elastic modulus possesses an almost linear increase with the increased content of nanoparticles (Figure 1a), but similarly to reports in literature a decline in tensile strength is inevitable.

### 3.2. Electrical Conductivity

**Figure 2:**
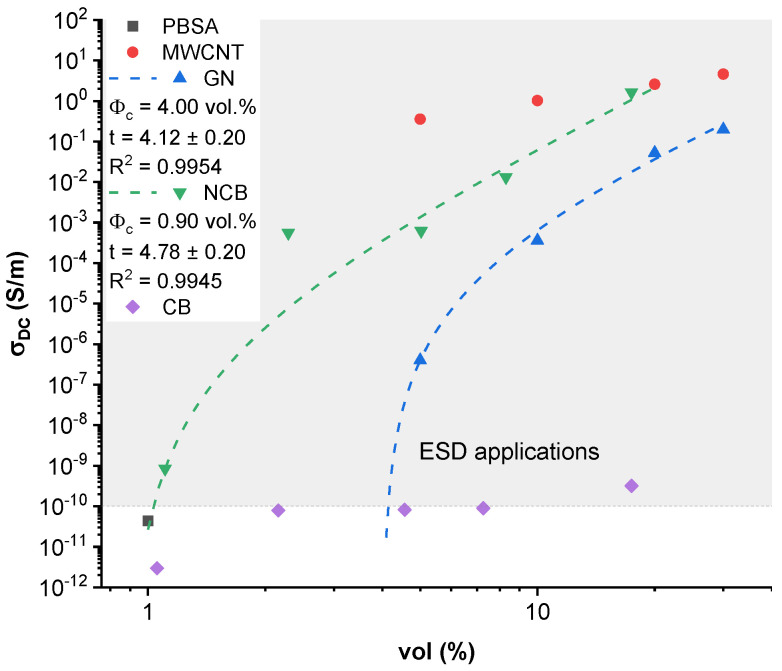
DC conductivity with log-log fit of PBSA/carbon nanoparticle composites.

The DC electrical conductivity of PBSA and its composites as a function of the volume content of nanoparticles is presented in Figure 2. PBSA has a measured conductivity of 4.37 × 10^−11^ S/m which is comparable to values observed by Ohki et al. (4.89 × 10^−11^ S/m) [16] and several magnitudes higher than comparable synthetic materials such as LDPE LDPE (~5 × 10^−13^ S/m) [57]. According to Tibbets et al., ESD materials require a minimum volume conductivity of 10^−10^ S/m [58]; therefore, PBSA is close to being suitable for ESD applications. The highest volume conductivities have been measured for 30.0 vol.% MWCNT (4.6 S/m) and 17.4 vol.% NCB (1.6 S/m) filled nanocomposites, indicating a trend that favors high loadings of fillers. A percolation threshold can be extrapolated for NCB-filled composites around the 0.9 vol.% concentration and GN composites around 4.0 vol.% (dashed line in Figure 2). The percolation threshold was approximated according to classic percolation theory [59] and the following equation [60]:

(6) $$\sigma_{DC}=\sigma_{0}{\left(\varphi -\varphi_{c}\right)}^{t}$$

where $\sigma_{DC}$ (S/m) is the composite DC conductivity, $\sigma_{0}$ (S/m) is the approximated filler conductivity, φ is the filler concentration, $\varphi_{c}$ is the percolation threshold, and t is the critical exponent describing the dimensionality of the system. For a 2D percolated system, t is approximately 1.33; for a 3D percolated system, t should be equal to 2 or more. To acquire a curve that would fit the experimental data, $\varphi_{c}$ was varied in small increments until a high R2 value could be achieved for experimental log($\sigma_{DC}$) over log$\left(\varphi -\varphi_{c}\right)$ data, as can be seen in Figure S2. The t exponent was acquired from the slope of the linear fit.

In CB-filled composites, relatively low improvements (3.2 × 10^−10^ S/m at 17.4 vol.%) or even a decrease (2.95 × 10^−12^ S/m at 1 vol.%) was observed compared to neat PBSA. A direct comparison at about 5.0 vol.% loading clearly shows that MWCNT is superior filler for the enhancement of electrical conductivity, yielding a ten order-of-magnitude improvement over CB, six orders over GN, and three orders over NCB.

Composite conductivity relates strongly with the aspect ratio and 3D structure of the carbon nanoparticles. In the case of CB, almost no influence of the filler can be seen at the given concentrations. This can be explained by the amorphous nature and globular shape (low packing factor) of CB nanoparticles, which are unable to form a network that aids electron conduction in the PBSA matrix. As for other three fillers, GN has a stiff lamellar structure and it necessitates higher filler loadings. NCB has a voluminous onion-like structure with a much higher specific surface area, thus allowing more contacts between nearby particles through contact conductivity or electron tunnelling [61,62]. MWCNT has a very high aspect ratio due to its tubular structure, allowing entanglements between individual nanotubes, thus facilitating the formation of a percolated structure. Regarding the previously mentioned $\sigma_{DC}$ specification for usage in ESD applications, all tested loadings for MWCNT, GN, NCB fillers and 17.4 vol.% CB loaded composite have volume conductivities above 10^−10^ S/m.

### 3.3. Surface Resistivity

**Figure 3:**
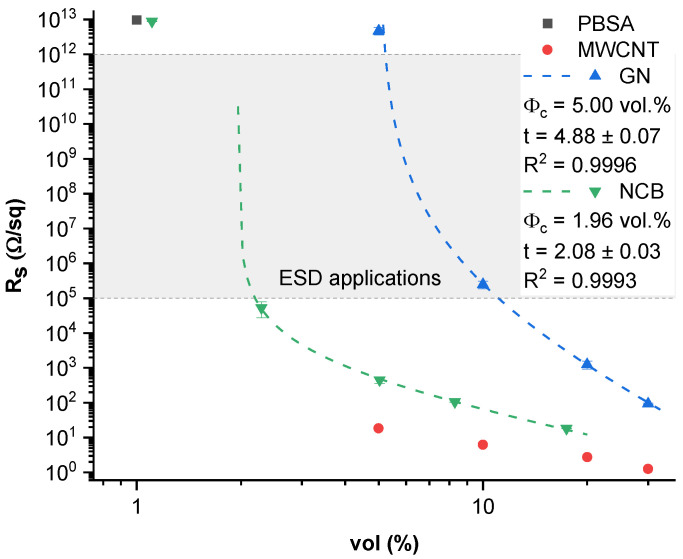
Surface resistivity of PBSA/carbon nanoparticle composites.

Figure 3 displays the surface resistivity values obtained from four-point probe measurements. The lowest surface resistivity values can be seen in the MWCNT-filled composites, achieving 1.26 Ω/sq at 30 vol.% loading. Comparatively, neat PBSA and CB-filled composites had an $R_{s}$ value higher than 10^13^ Ω/sq (above the measurable range of used equipment). An electrical conductivity percolation threshold can be observed between 5–10 vol.% for GN and between 1.1 and 2.2 vol.% for NCB loaded composites, which is highly dependent on the aspect ratio of nanoparticles, as discussed before. The surface resistivity percolation thresholds (dashed line, Figure 3) were approximated similarly to DC conductivities according to Equation (6). The tested MWCNT composites are obviously above the percolation threshold due to the high loading level selected. According to Pötschke et al., the electrical percolation threshold in MWCNT (NC7000) melt-mixed composites can be observed at rather low concentrations of between 0.175–0.26 vol.% [63].

According to the US Department of Defense performance specification for ESD barrier materials [64], packaging materials used for the inner layer require $R_{s}$ between 10^5^ to 10^12^ Ω/sq, and for the outer layer $R_{s}$ should be less than 10^12^ Ω/sq. Evaluating the tested composites for potential application as ESD inner layer material, only the 10 vol.% filled GN composite falls within the required $R_{s}$ (2.48 × 10^5^ Ω/sq) specification, while 2.2 vol.% filled NCB composite is almost suitable (5.31 × 10^4^ Ω/sq). For use in ESD applications, it would be necessary to increase the surface resistivities of NCB, GN and MWCNT composites, which would be possible by choosing concentrations around the percolation threshold. NCB and MWCNT would be more effective for this application, as a smaller loading of fillers is required to achieve electrical percolation. Our tested CB is not suitable for ESD applications, as very high filler loadings would be necessary to decrease surface resistivity to an appropriate extent. In case of ESD outer layer materials, electrically percolated composites or composites at percolation threshold are suitable, and most of the tested composites are usable for this application.

### 3.4. Thermal Conductivity

**Figure 4:**
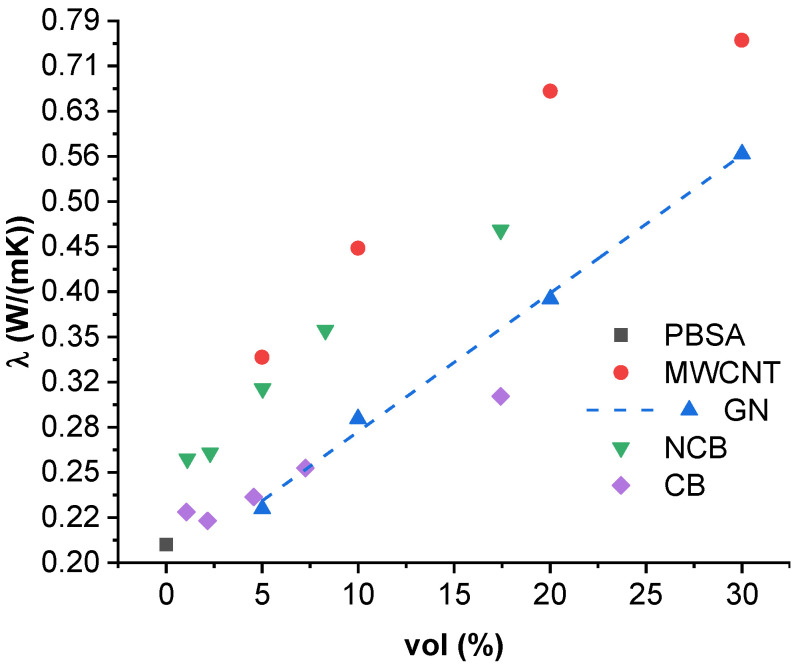
Thermal conductivity of PBSA/carbon nanoparticle composites in logarithmic scale at 25 °C.

Figure 4 shows thermal conductivity values of PBSA composites at 25 °C, while Figure S3 show conductivities at 35 and 45 °C. As thermal conductivity is a thermally activated process (further discussed in Section 3.5.), the highest thermal conductivities were achieved at 45 °C. The highest thermal conductivity values at 25 °C were reached for the MWCNT-filled composites, with 30.0 vol.% filled composition reaching 0.756 W/mK (262% increase over PBSA), followed by 20.0 vol.%, 10.0 vol.% and 5.0 vol.% compositions, which showed an increase of 218%, 113% and 61% over neat PBSA. GN-filled systems show a maximum thermal conductivity of 0.566 W/mK (171% increase over PBSA) with 30.0 vol.% loading. The change in thermal conductivity with increase in GN loading can be described by the proportionality $\lambda \propto \exp\left(\Phi_{f}\cdot a-b\right)$, where $\Phi_{f}$ is the filler content, and a (0.0153) and b (−0.7081) are fitted approximation constants (Figure 4 dash line). For NCB-filled systems, the highest thermal conductivity was observed for 17.4 vol.% loaded composite, at 0.466 W/mK (123% increase over PBSA). The lowest thermal conductivity increase can be seen in the CB-filled systems, reaching only 0.305 W/mK (46% increase) for the 17.4 vol.% loading. Comparing the volume specific thermal conductivity improvement (%_improvement_/vol.%), the most effective filler is NCB, reaching 22.06%/vol.% with a 1.1 vol.% loading, and 11.48%/vol.% for the 2.3 vol.% loading. The next most efficient filler is MWCNT, which shows 12.27%/vol.% for the 5.0 vol.% loading.

Thermal conductivity in solids is attributed to the transport of heat carriers, which can be electrons and phonon quasiparticles (the manifestation of elastic vibration in the atomic or molecular lattice) [65]; for polymers and composites, phonon conductivity vastly dominates over electronic conductivity. In the case of PBSA and carbon nanoparticle composites, the increase in thermal conductivity is attributed to two main factors: First, due to the increase in the concentration of free dislocated electrons, and the formation of a percolated network through which electrons can flow with reduced resistance and tunneling. Second, reduced phonon resistance, mainly due to inclusion of more structured and highly conductive fillers (covalently bonded carbon), and the previously mentioned percolated network. The main mechanism of thermal transport in semi-crystalline polymers is contact conductivity between separate macromolecules under thermal vibration, as well as conductivity through highly ordered and highly conductive crystalline regions [66]. There is also the third factor influencing the thermal conductivity of PBSA systems, which is the effect of increased polymer crystallinity (and thus decreased phonon resistance) due to heterogenous nucleation caused by filler inclusions [67]. However, it is not discussed in the present paper.

The thermal conductivity of the composites is highly dependent on the nature of the filler particles. More crystalline fillers, such as MWCNT, GN and NCB, create less phonon resistance. High surface areas and aspect ratios allow the formation of a dense and percolated 3D structure, as is the case with MWCNT and NCB. While GN has high reported conductivities in the in-plane direction, the conductivity normal to the plane is comparable to regular graphite [68]; therefore, the biggest contributions to thermal conductance would be contact through the edges of the nanoplatelets. The platelet shape can also contribute to a percolated structure, but at higher filler loadings than the fibrous structure of MWCNT and onion-like structure of NCB. The thermal conductivity might also be influenced by the size of the nanoparticles through the inverse proportionality by increasing the average particle size, the number of interfaces and discontinuities of crystal lattice that a given phonon would need to traverse are reduced and vice versa [69]. The influence of filler–matrix interaction on the thermal conductivity of a polymer composite has been discussed in length in a paper by Han et al., in the case of a composite of graphene and a styrene-based copolymer [70].

### 3.5. Thermal Conductivity Activation Energy

**Figure 5:**
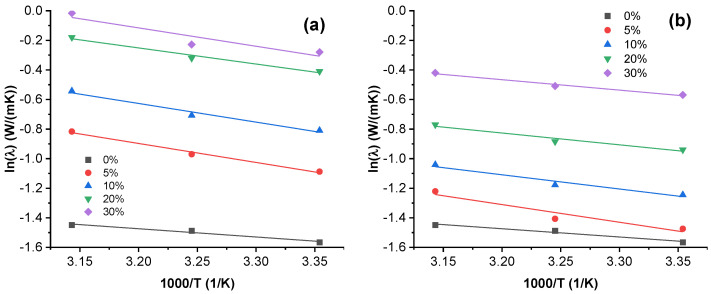
Arrhenius plots of thermal conductivity dependence on temperature for (**a**) MWCNT composites and (**b**) GN composites.

Thermal conductivity Arrhenius plots are presented in Figure 5 for MWCNT and GN composites. Data for NCB and CB composites are shown in Supplementary Material (Figure S4). All the tested composites display temperature-dependent thermal conductivity. Table 2 shows the activation energies $E_{a}$ and inherent thermal conductivity $\lambda_{0}$ values according to the Arrhenius Equation (5). $E_{a}$ is the minimum energy necessary for a polymer composite system to overcome a thermally activated potential barrier. $\lambda_{0}$ is the inherent thermal conductivity at infinite temperature. NCB, MWCNT and GN composites show a trend of decreasing activation energy with an increase in filler loading. A neat PBSA system displays activation energy of 0.48 eV and inherent conductivity of 1.36 W/(mK). The thermal dependence in polymer systems can be attributed to the temperature-dependent mobility of macromolecules causing more frequent contacts and phonon transfers between separate macromolecules [71]. The addition of any filler greatly increases the thermal dependence, which can be explained by the introduction of thermally activated carriers (electrons) or a change in the polymer structure (crystallinity). The nonmonotonic behavior of $\lambda_{0}$ and $E_{a}$ could be caused by the formation of defects, e.g., agglomeration and cracks, or due to the formation of a very dense filler–filler network, where contact conductivity dominates over electron tunneling. The massive difference in filler loading from 1.1 to 30.0 vol.% contributes to different structural arrangements in the composite material, which make the comparison complicated without an in-depth analysis of the structure. The lowest increase can be seen in the CB composites, while the highest temperature dependence can be seen for the 5.0 vol.% MWCNT composite with a value of 0.111 eV. The decrease in the temperature dependency can be explained by the development of a denser percolated structure, allowing shorter electron tunneling distances and easier electron transport. The greatest decrease in temperature dependency with an increase in filler volume is seen in the NCB composites (from 0.098 eV at 1.1 vol.% to 0.047 eV at 17.4 vol.%). The inherent thermal conductivity is dependent both on the available thermally activated carriers, as well as the distribution and dispersion of the filler particles, as well as the interaction between the filler and the polymer matrix, with carriers flowing through paths of least resistance. MWCNT provides both free electrons as well as continuous paths, while in the case of GN, NCB, and CB, the path length is likely to be increased.

### 3.6. Microwave Spectroscopy and Terahertz Transmittance

**Figure 6:**
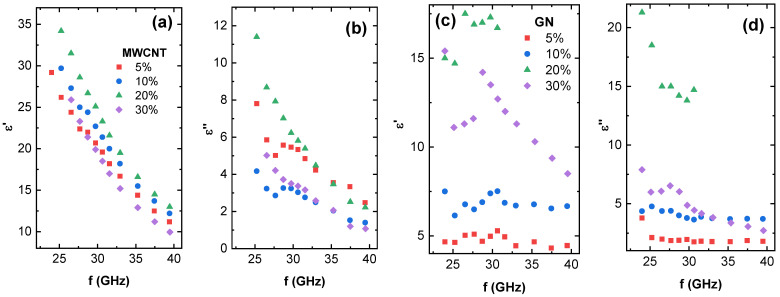
Microwave dielectric spectroscopy (**a**) real part of permittivity for MWCNT composites, (**b**) imaginary part of permittivity for MWCNT composites, (**c**) real part of permittivity for GN composites, and (**d**) imaginary part of permittivity for GN composites.

Dielectric properties of the composites in the microwave spectrum frequency range from 24 to 40 GHz are shown in Figure 6 and Figure S5. Two different separate dielectric responses can be observed. For all MWCNT composites, 20–30 vol.% GN composites, and 4–7 vol.% NCB composites, a frequency-dependent decrease in both real and imaginary parts of dielectric permittivity can be seen, in good accordance with the Jonsher universal law [72]. The highest real permittivity is observed for MWCNT composites. However, the highest imaginary part of permittivity was observed in 20 vol.% GN and 7.3 vol.% NCB composites. A frequency-independent permittivity response can be seen in the 5.0–10.0 vol.% GN and 1.1–2.3 vol.% NCB samples, which correlates with their lower electrical conductivity as seen in Figure 2 and lower density of the percolated network. The achieved dielectric permittivity values are in line with values obtained in other thermoplastic carbon-nanoparticle composites in literature [73], and therefore our developed composites have the potential to be used for EMI shielding applications. Moreover, 1 mm thick plates of composites filled with 20 vol.% GN and 7.3 vol.% NCB exhibit more than 54% absorption at 25 GHz, while the microwave absorption of corresponding plate composites filled with MWCNT is slightly less than 50% [74]. This denotes that in the current matrix GN and NCB are more effective fillers for electromagnetic applications than MWCNT.

**Figure 7:**
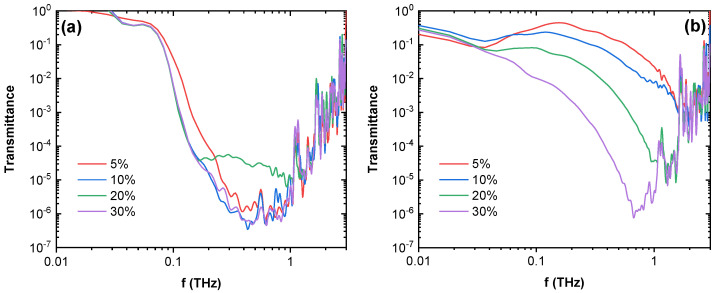
Time-domain spectroscopy transmittance spectra for (**a**) MWCNT and (**b**) GN composites.

The time-domain spectroscopy transmittance spectra for composites can be seen in Figure 7 for MWCNT and GN composites and Figure S6 for CB and NCB composites. Composites filled with MWCNT, GN and NCB show a drop in transmittance by up to six orders of magnitude, effectively becoming opaque at higher frequencies (above 1 THz for NCB composites, 700 GHz for GN composites, and 400 GHz for MWCNT composites), compared to CB-filled composites, which displayed a decrease of only up to a single order. MWCNT-filled composites show a rapid decrease in transmittance from 0.03 THz to approximately 1 THz, reaching a transmittance minimum, where a linear frequency dependent region begins and continues up to approximately 3 THz, which is an artifact, related to the absence of both transmitted and reference signals. MWCNT composites, except for the 20.0 vol.%, show similar spectra and similar values of transmission, while the 20.0 vol.% composite shows a plateau at a higher transmittance, which could be related to structural defects in the sample. GN and NCB composites show similar behavior; however, there is a higher degree of transmittance dependence on filler concentration, with the lowest transmittance seen in the highest loadings. This is in good agreement with the DC conductivity and microwave dielectric property behavior of composites (Figure 2 and Figure 6). In the terahertz frequency range, it is also expected that the complex dielectric permittivity of composites changes slowly with MWCNT concentration (concentrations are far above the percolation threshold), while with GN and NCB the change is sudden (concentrations are close to the percolation threshold). GN and NCB composites also display a narrower minimum peak that moves towards higher frequencies.

## 4. Conclusions

The present study serves to elucidate the potential of PBSA within the fields of anti-static packaging, electrostatic dissipative, and electromagnetic interference shielding materials, allowing the further proliferation of biodegradable plastics, as well as modern nanoparticle-filled composites.

Electrical percolation is observed in composites filled with MWCNT, GN and NCB inclusions at concentrations less than 10.0 vol.%. The lowest values of the percolation threshold are typical for composites with MWCNT and NCB nanoparticles. Composites with MWCNT, GN and NCB inclusions above the percolation threshold are suitable for electromagnetic shielding applications in microwave and THz frequency ranges. Most promising are composites with NCB inclusions, where even for concentrations of 7.2 vol.%, above 50% microwave shielding can be obtained.

The addition of carbon nanofillers served to significantly improve the elastic modulus, with the higher filler loading contributing to a greater increase. MWCNT composites showed the highest increase in elastic modulus, a 79% to 266% increase over PBSA. Carbon nanoparticles showed excellent compatibility with the PBSA matrix by enhancing tensile strength with filler loadings up to 5 vol.% (the highest increase (21%) was observed for 2.2 vol.% NCB) while MWCNT retained enhanced tensile strength with loadings up to 20 vol.%. Composites with filler loading up to 5 vol.% retained yield strain values above 10%, while filler loading between 5 to 10 vol.% had yield strain values above 5%. The higher loadings above 10 vol.% significantly affected yield strain values, making samples relatively stiff and fragile, except for 20 vol.% MWCNT, which retained a yield strain value of 4.6%. Thus, nanocomposites with high filler loadings might not be suitable for high-strain dynamically stressed applications but could still be used for more statically stressed parts and in fields where the added electrical, thermal, ESD and EMI properties are more important than mechanical properties (printed circuit board components, heat sinks, EMI shielding). Nanocomposites with lower filler loadings are suitable for use in ESD-compliant soft packaging.

For future research the authors see several routes to expand upon this topic including comparing different preparation methods and their impact on composite properties, focusing research on one specific application with confined loading distribution, and in-depth study of the nanoparticle impact on crystallinity and phase formation.

## Figures and Tables

**Table 1:** Prepared compositions of PBSA composites and measured density values.

Nr.	Filler	vol.%	wt.%	Density (g/cm^3^)
1	-	-	-	1.2272 ± 0.0014
2	MWCNT	5.0	7.3	1.2623 ± 0.0021
3		10.0	14.3	1.2894 ± 0.0109
4		20.0	27.4	1.3621 ± 0.0023
5		30.0	39.2	1.4136 ± 0.0096
6	GN	5.0	8.4	1.2630 ± 0.0017
7		10.0	16.1	1.2923 ± 0.0045
8		20.0	30.2	1.3681 ± 0.0175
9		30.0	42.6	1.4400 ± 0.0042
10	NCB	1.1	1.5	1.2321 ± 0.0017
11		2.2	3.2	1.2410 ± 0.0017
12		5.0	7.2	1.2571 ± 0.0025
13		8.3	11.7	1.2745 ± 0.0007
14		17.4	23.6	1.3268 ± 0.0122
15	CB	1.1	1.5	1.2198 ± 0.0010
16		2.2	3.1	1.2335 ± 0.0014
17		4.5	6.5	1.2465 ± 0.0010
18		7.2	10.3	1.2595 ± 0.0010
19		17.4	23.6	1.3097 ± 0.0015

**Table 2:** Parameters of the Arrhenius equation for PBSA composites.

Filler	Vol.%	$\lambda_{0}$ (W/mK)	$E_{a}$ (eV)
-	-	1.36	0.048
MWCNT	5.0	24.77	0.111
	10.0	30.17	0.109
	20.0	25.36	0.094
	30.0	45.79	0.105
GN	5.0	12.31	0.103
	10.0	7.14	0.083
	20.0	5.62	0.069
	30.0	5.99	0.061
NCB	1.1	11.90	0.098
	2.2	10.65	0.095
	5.0	4.76	0.070
	8.3	4.46	0.064
	17.4	2.89	0.047
CB	1.1	2.30	0.059
	2.2	3.72	0.072
	4.5	2.26	0.058
	7.2	3.47	0.068
	17.4	7.03	0.081

## Data Availability

The data presented in this study are available on request from the corresponding author.

## Supplementary Materials

The following supporting information can be downloaded at: https://www.mdpi.com/article/10.3390/nano12203671/s1, Figure S1: Stress strain curves of (a) MWCNT (b) GN (c) NCB (d) CB, Figure S2: Approximation fits for electrical percolation threshold of GN and NCB DC conductivity, Figure S3: Thermal conductivity values at (a) 308.15 K (b) 318.15 K, Figure S4: Arrhenius plots of thermal conductivity dependence on temperature for (a) NCB (b) CB, Figure S5: NCB microwave dielectric spectroscopy (a) real part of permittivity (b) imaginary part of permittivity, Figure S6: Time domain spectroscopy transmittance spectra of composites (a) NCB (b) CB.
