# Supplementary material for: Comparison of Carbon-Nanoparticle-Filled Poly(Butylene Succinate-co-Adipate) Nanocomposites for Electromagnetic Applications
Source: Nanomaterials (Basel). 2022 Oct 19;12(20):3671. doi: 10.3390/nano12203671 (PMC9609898; doi:10.3390/nano12203671)
Supplement: Supplementary file 1 [file nanomaterials-12-03671-s001.zip › nanomaterials-1976337-supplementary.pdf]

Supplementary Materials

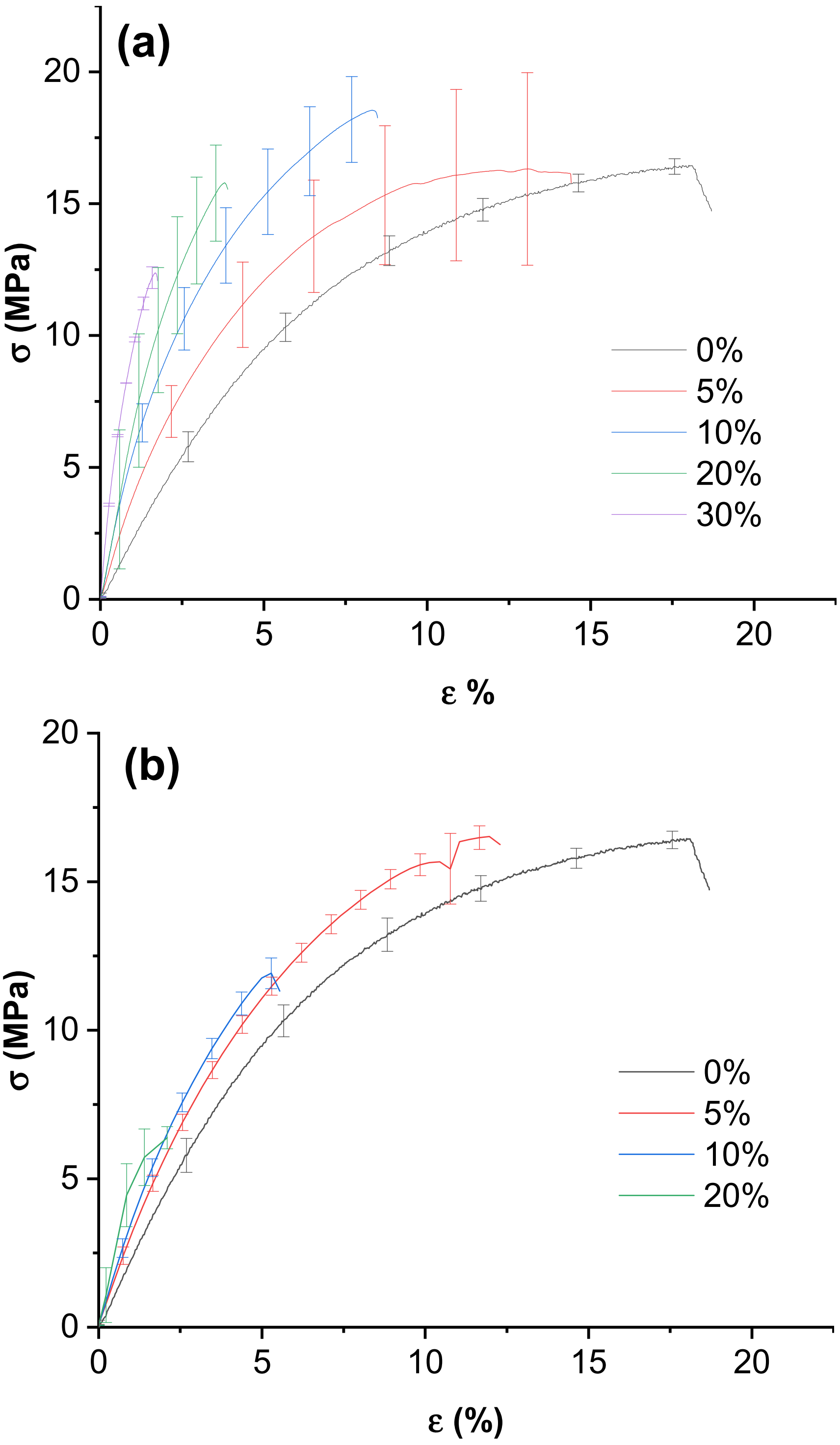

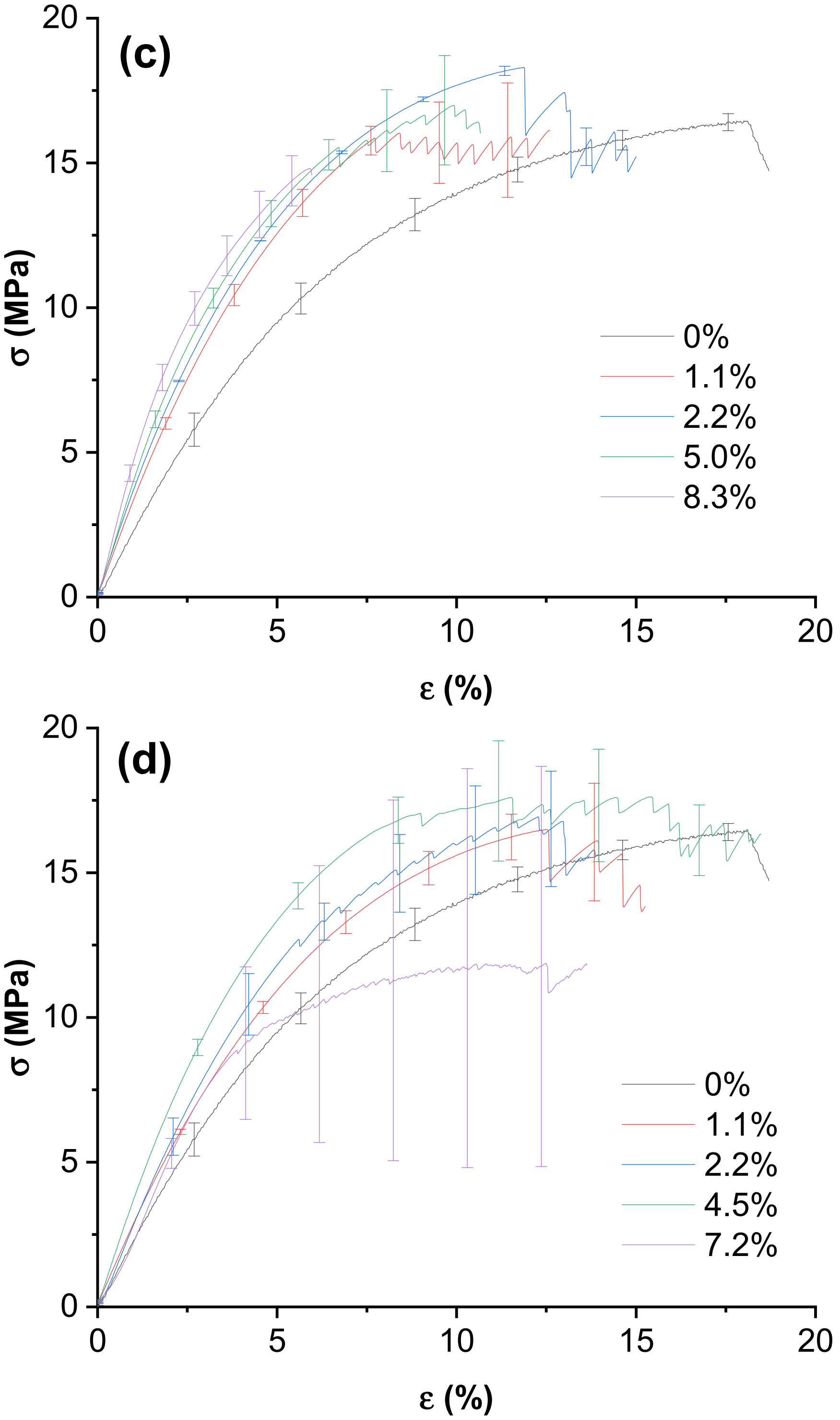

Figure S1. Stress strain curves of (**a**) MWCNT, (**b**) GN, (**c**) NCB, and (**d**) CB.

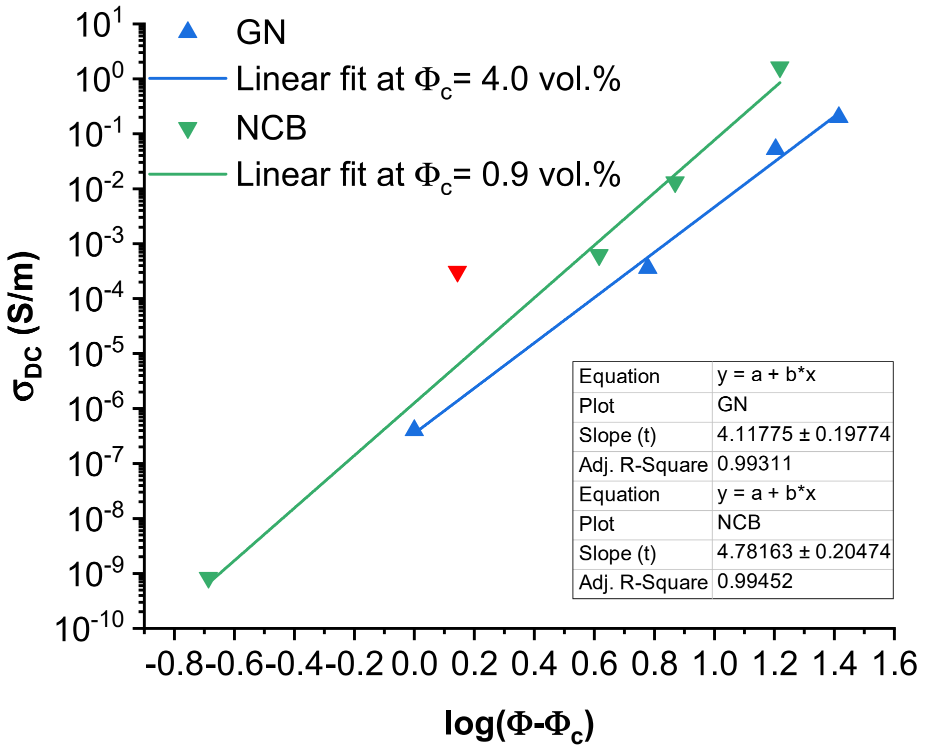

Figure S2: Approximation fits for electrical percolation threshold of GN and NCB DC conductivity.

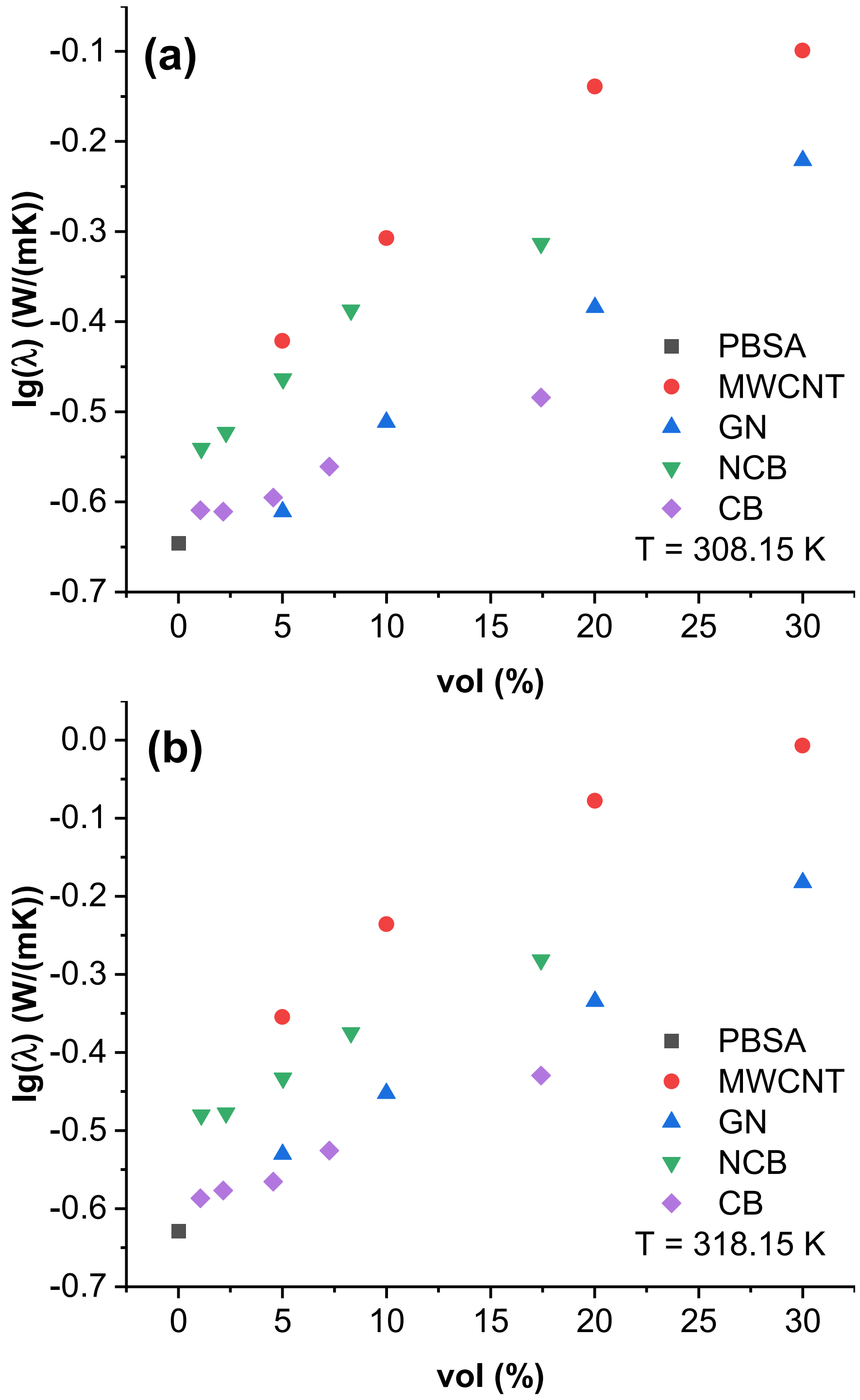

Figure S3: Thermal conductivity values at (**a**) 308.15 K and (**b**) 318.15 K.

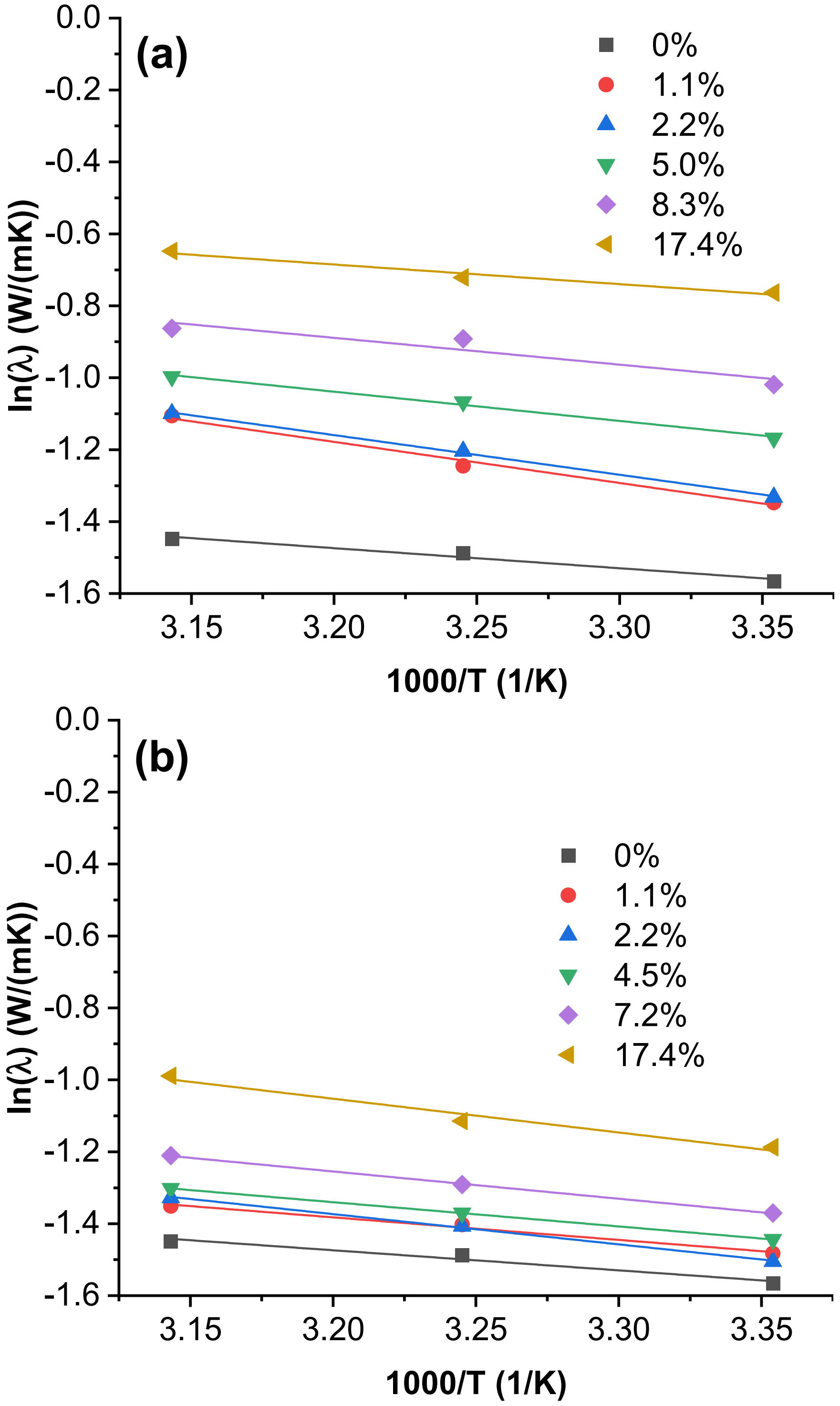

Figure S4: Arrhenius plots of thermal conductivity dependence on temperature for (**a**) NCB and (**b**) CB composites.

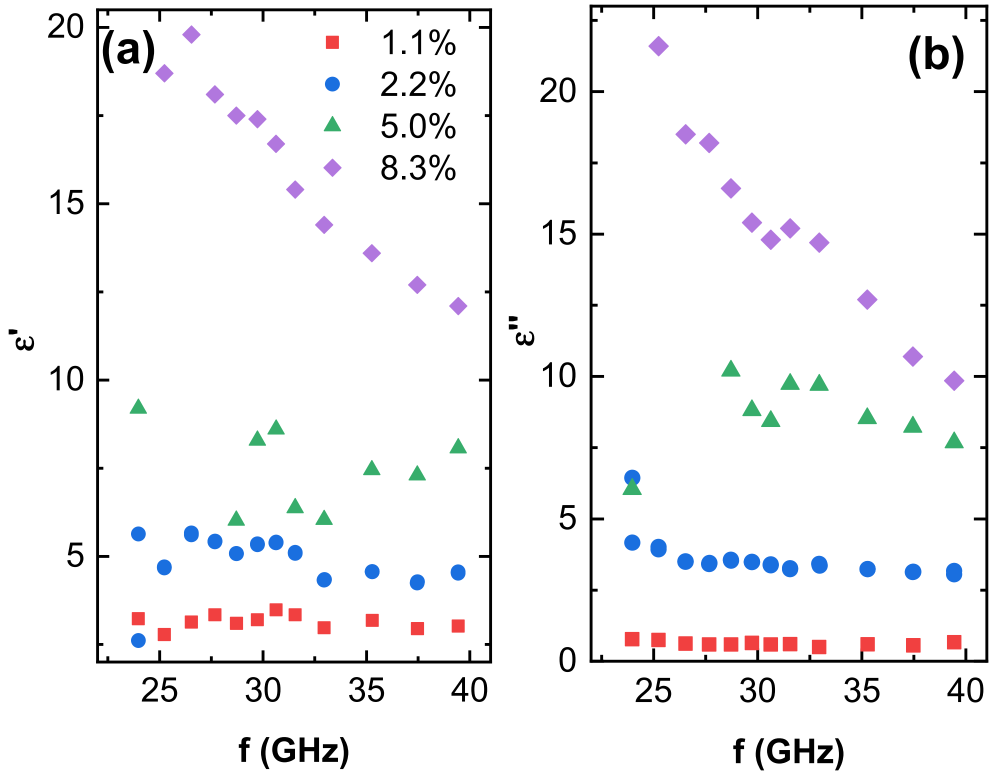

Figure S5: NCB composite microwave dielectric spectroscopy (**a**) real part of permittivity and (**b**) imaginary part of permittivity.

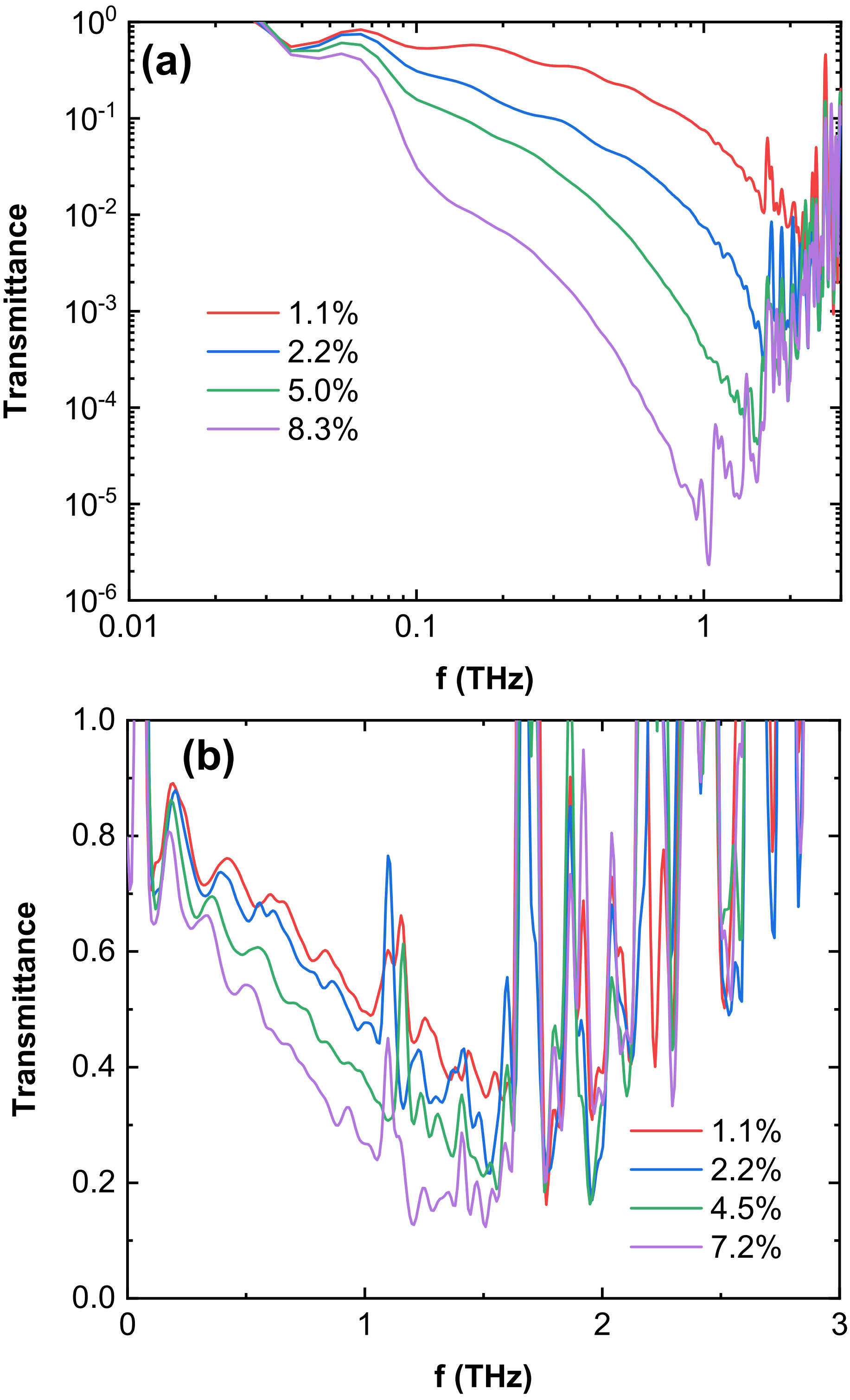

Figure S6: Time domain spectroscopy transmittance spectra of composites with (**a**) NCB and (**b**) CB fillers.
